# XGBOOST MACHINE LEARNING TO IDENTIFY PREDICTIVE VALUES OF CARDIOMETABOLIC RISK FOR COGNITIVE DECLINE AND MORTALITY

**DOI:** 10.1093/geroni/igae098.3293

**Published:** 2024-12-31

**Authors:** Longjian Liu, Saishi Cui, Ruifeng Wang, Kaidi Xu, Howard Eisen

**Affiliations:** Drexel University, Philadelphia, Pennsylvania, United States; Drexel University, Philadelphia, Pennsylvania, United States; Drexel University, Philadelphia, Pennsylvania, United States; Drexel University, Philadelphia, Pennsylvania, United States; Thomas Jefferson University Hospital, Philadelphia, Pennsylvania, United States

## Abstract

**Aim:**

To investigate the crucial biomarkers of cardiometabolic disorders that contribute to the risk of cognitive decline and mortality among adults with cognitive decline and dementia.

**Methods:**

We analyzed a cohort of 6814 participants aged 45 to 84 years at the baseline of 2000-2002 in the Multi-Ethnic Study of Atherosclerosis. Cognitive function was assessed in 4,591 during 2010-2012. Dementia included Alzheimer’s Disease (AD) and AD-related dementia. All participants were followed through to December 2015. XGBoost based machine learning (ML) approach, a novel technique, was used to identify the important features of various biomarkers for assessing the risk of cognitive decline and mortality in those with cognitive decline and dementia.

**Results:**

Among 5723 with valid follow-up information, 227 died in those with cognitive decline or dementia. The mortality rate (95%CI) was 3.8 (3.2-4.5) per 1000 person-years in males, and 2.1 (1.7-2.5) per 1000 person-years in females. XGBoost models identified the top five critical biomarkers: carotid intimal-medial thickness, elevated glucose, urinary albumin to creatine ratio (UACR), interleukin 6, and factor VIII. The XGBoost models demonstrated an accuracy, sensitivity, specificity, and area under the receiver operating characteristic curve (AUC) of 74%, 73%, 75%, and 85%, respectively, in classifying cognitive decline. XGBoost survival models identified top five biomarkers: CIMT, UACR, homocysteine, cystatin-C, and D-Dimer for mortality risk, with a C-index of 0.86% and a mean time-dependent AUC of 90%. Conclusions: XGBoost based ML approaches effectively identified the pivotal features among multiple biomarkers of cardiometabolic disorders linked to the risk of cognitive decline and mortality.

